# Small differences in root distributions allow resource niche partitioning

**DOI:** 10.1002/ece3.6612

**Published:** 2020-08-26

**Authors:** Andrew Kulmatiski, Karen H. Beard, Martin C. Holdrege, Edmund C. February

**Affiliations:** ^1^ Department of Wildland Resources and the Ecology Center Utah State University Logan UT USA; ^2^ Department of Botany University of Cape Town Cape Town South Africa

**Keywords:** ecohydrology, hydrus 1D, isotope, niche partitioning, tracer, two‐layer hypothesis, water use

## Abstract

1. Deep roots have long been thought to allow trees to coexist with shallow‐rooted grasses. However, data demonstrating how root distributions affect water uptake and niche partitioning are uncommon.
2. We describe tree and grass root distributions using a depth‐specific tracer experiment six times over two years in a subtropical savanna, Kruger National Park, South Africa. These point‐in‐time measurements were then used in a soil water flow model to simulate continuous water uptake by depth and plant growth form (trees and grasses) across two growing seasons. This allowed estimates of the total amount of water a root distribution could absorb as well as the amount of water a root distribution could absorb in excess of the other rooting distribution (i.e., unique hydrological niche).
3. Most active tree and grass roots were in shallow soils: The mean depth of water uptake was 22 cm for trees and 17 cm for grasses. Slightly deeper rooting distributions provided trees with 5% more soil water than the grasses in a drier season, but 13% less water in a wetter season. Small differences also provided each rooting distribution (tree or grass) with unique hydrological niches of 4 to 13 mm water.
4. The effect of rooting distributions has long been inferred. By quantifying the depth and timing of water uptake, we demonstrated how even small differences in rooting distributions can provide plants with resource niches that can contribute to species coexistence. Differences in total water uptake and unique hydrological niche sizes were small in this system, but they indicated that tradeoffs in rooting strategies can be expected to contribute to tree and grass coexistence because 1) competitive advantages change over time and 2) plant growth forms always have access to a soil resource pool that is not available to the other plant growth form.

## Graphical abstract

Plant water uptake is often inferred from root biomass, but here we use a tracer study and water flow model to estimate plant water uptake by depth for trees and grasses. Results show how even small differences in rooting depths can create resource niches that can allow species coexistence.

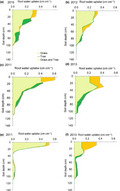

## 1 INTRODUCTION

It has long been recognized that plant species have different vertical rooting patterns (Ward, Wiegand, & Getzin, 2013). It is likely that these differences affect water and nitrogen uptake and partitioning, and therefore plant growth and coexistence (Walker & Noy‐Meir, 1982). Defining vertical niche partitioning is a long‐standing interest of savanna ecologists (Walker & Noy‐Meir, 1982; Ward et al., 2013). For over 100 years, Walter's two‐layer hypothesis has suggested that deep roots allow trees to grow even in the presence of shallow‐rooted grasses that have preferential access to precipitation (Ward et al., 2013). Soil N concentrations also tend to be greatest near the surface, so shallow roots may also provide preferential access to soil N. Implicit in this two‐layer hypothesis is that shallow grass root mats are competitively superior for capturing soil water and N. Yet, it remains difficult to measure the amount of water or N that different plant species absorb under field conditions (Dubbert & Werner, 2019). Without measurements of resource uptake, these hypotheses remain poorly tested (Holdo, 2013; Silvertown, Araya, & Gowing, 2015; Smithwick, Lucash, McCormack, & Sivandran, 2014).

Our understanding of niche partitioning is often inferred from root biomass distributions and not measurements of resource uptake (Grant & Dietrich, 2017; Sala, Lauenroth, & Parton, 1992). Yet, there is reason to believe root biomass distributions provide poor inference for resource uptake (Kulmatiski, Adler, Stark, & Tredennick, 2017; Schymanski, Sivapalan, Roderick, Beringer, & Hutley, 2008). Root biomass data is typically biased by the presence of large, relatively heavy suberized roots that do not actively absorb soil resources and are more common near the stem and soil surface (Nippert & Holdo, 2015; Barberon et al., 2016). Even fine roots do not absorb water from dry soils (e.g., when soil water potential < −3 MPa; Gambetta, Knipfer, Fricke, & McElrone, 2017; Schenk, 2008), and fine roots can vary widely in their ability to absorb soil resources (Hodge, 2004; McKane et al., 2002). Thus, techniques that account for both the presence of active roots and resource availability are needed (Schymanski et al., 2008; da Silva et al., 2011).

Stable isotope techniques can integrate the effects of root presence, root activity, and resource availability (van der Heijden et al., 2015; Rothfuss & Javaux, 2017). There are two broad classes of stable isotope techniques used to describe soil resource uptake: natural abundance and pulse‐chase approaches. Natural abundance approaches do not require site preparation allowing spatial and temporal replication and broad inference. Natural abundance approaches, however, require naturally occurring isotope gradients, which often provide only broad inference, such as groundwater versus vadose water, or water use greater than 50 cm or less than 50 cm (Dubbert & Werner, 2019; Ogle & Reynolds, 2004; Rothfuss & Javaux, 2017).

Pulse‐chase approaches can provide a very detailed description of vertical and horizontal resource uptake even at depths greater than 50 cm (Beyer et al., 2016; Mamolos, Elisseou, & Veresoglou, 1995; McKane et al., 2002; Sternberg, 2002). However, injections often are not performed over large areas. Injections can also result in species‐bias due to differences in plant size or rooting zone, though this can be addressed by comparing the proportion of tracer uptake among species (Kulmatiski, Beard, Verweij, & February, 2010). Finally, because they introduce tracer water, injections may detect active roots in otherwise dry soils. This can be controlled by using tracer‐defined rooting profiles in soil water movement models that account for water availability (Mazzacavallo & Kulmatiski, 2015). The use of soil water flow models has the additional benefit of providing continuous estimates of depth‐ and species‐specific water use (Holdo & Nippert, 2015; Kulmatiski, Adler, & Foley, 2020; Zheng et al., 2018).

Here we use a pulse‐chase experiment to describe vertical patterns of hydrologic tracer uptake by trees and grasses at three times during each of two growing seasons in a subtropical savanna in Kruger National Park, (KNP) South Africa. These point‐in‐time measurements were used in a soil water flow model to simulate continuous water uptake for tree and grass rooting distributions separately. The only factor to change between simulations of tree and grass water uptake was the rooting distribution. This approach produced estimates of the total amount of water a rooting distribution could extract across depths and time (i.e., total water uptake), and identified the amount of water each rooting distribution could extract in excess of the other at particular depths or times (i.e., a unique hydrological niche). Because nitrogen is a common limiting nutrient, the uptake of which is also determined by plant rooting distributions, we also describe vertical patterns of soil nitrogen uptake by injecting ^15^N with water tracers during one mid‐season sampling. This provided a test of whether or not N uptake patterns for trees and grasses differed from water uptake patterns (Kulmatiski et al., 2017; McKane et al., 2002; da Silva et al., 2011). We predicted that root distributions would help explain tree and grass coexistence because differences in rooting distributions would result in tradeoffs that provide either trees or grasses more water or better access to soil water at certain depths and times as a function of water infiltration into the soil.

## 2 METHODS

### 2.1 Study site

**FIGURE 1:**
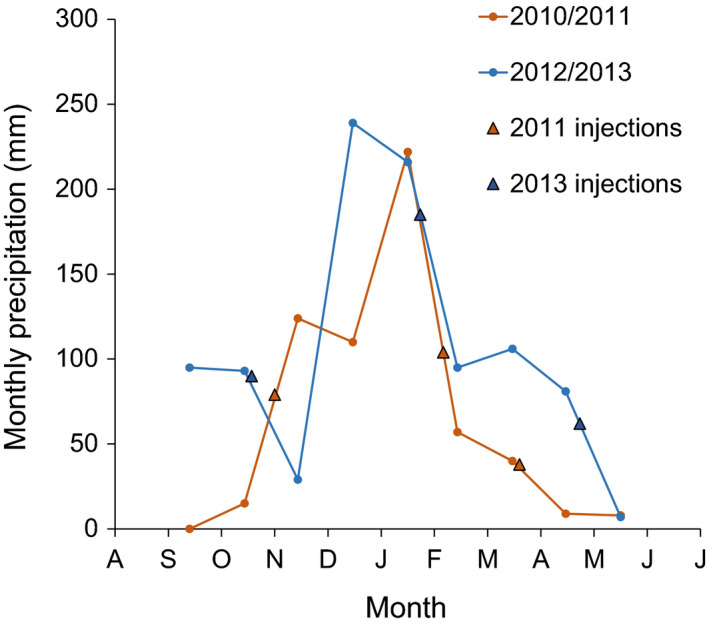
Precipitation patterns and sampling times. Long‐term mean monthly precipitation (black hashed line) and monthly precipitation during the 2010/2011 growing season (red line), and the 2012/2013 growing season (blue line). Triangles indicate sampling dates

Research was conducted in a subtropical savanna on clay soils during the 2010/2011 and 2012/2013 growing seasons, Lower Sabie, KNP, South Africa 25°12ʹ09.34ʺS 31°54.28ʺE; 191 m elevation). In contrast to two similar previous studies that were performed in mesic/sandy conditions (Kulmatiski et al., 2010) and xeric/clay conditions (Mazzacavallo & Kulmatiski, 2015), this study was performed in mesic/clay conditions. Soils are smectic clays derived from the underlying basaltic bedrock (Buitenwerf, Kulmatiski, & Higgins, 2014; Venter, Scholes, & Eckhardt, 2003). The climate is typified with a cool–dry season (June–October) and a hot–wet season (November–May). Mean annual growing season precipitation (October through May) is 618 mm. Precipitation during the two study seasons was 587 and 866 mm, respectively (Figure 1). Average winter (June–November) and summer temperatures are 19.6°C and 23.6°C, respectively. During the 2010/2011 and 2012/2013 growing seasons, average temperatures were 23.6°C and 23.3°C, respectively.

### 2.2 Tracer experiment

Methods for our tracer experiment and water modeling are outlined in Kulmatiski et al. (2020). At the beginning of the 2010/2011 growing season, a grid with 63 points, each separated by 15 m was established across the study site. During each of the early‐, mid‐ and late‐season samplings (Figure 1), 21 plots (7 m^2^ circles) were randomly assigned to a point in the grid. Three replicate plots were assigned to each of six soil depths (5, 10, 20, 30, 50, and 100 cm). The remaining three plots did not receive injections and were used to collect “control” samples (Kulmatiski et al., 2010; Mazzacavallo & Kulmatiski, 2015). Due to time constraints, the 30 cm plots were only sampled in the November 2011 sampling. Throughout each 7 m^2^ plot, a 15 cm by 15 cm grid with 314 points was established. At each point, a 13 mm‐wide, carbide‐tipped drill bit (Relton Corporation) on a hammer drill (TE‐60, Hilti North America) was used to create a hole to the target depth into which 3 ml syringes glued to hypodermic tubing (16 gauge thin‐walled hypodermic tubing; Vita Needle Company) were placed. In each of the 314 holes in each plot, 1 ml of 70% ^2^H_2_O (Cambridge Isotope Laboratories), followed by 2 ml of tap water rinse was injected (Kulmatiski et al., 2010). While injections likely resulted in a temporary and localized increase in plant available water at the point of injection, the 940 ml of water added to each 7 m^2^ plot represented <5% of daily transpiration in the plot and so were not expected to stimulate plant growth (Kulmatiski et al., 2020; Ramoelo et al., 2014).

Two days after injections, two to four samples of nontranspiring tissue from one to several individuals were collected from all common (i.e., >5% of community composition) species. For grasses, this was nongreen tissue within 1 cm of the root crown. For trees and shrubs, this was twigs below transpiring tissues. Assuming sap flow rates of 50–100 cm per day (Gifford, 1968; Scott, Cable, & Hultine, 2008), it is likely that the stem water we collected was absorbed by roots the previous day. As a result, our stem water isotope concentrations are assumed to represent water uptake over the one day following injections. Similarly, there was only one day for water to move from the injection point to nontarget depths (e.g., as a result of infiltration, evaporation, or hydraulic redistribution). Previous studies have found that the injected tracer is typically isolated to be within 10 cm of target injection depths (Berry & Kulmatiski, 2017; Kulmatiski et al., 2010; Mazzacavallo & Kulmatiski, 2015; Warren, Kulmatiski, & Beard, 2015). We collected samples using clippers that were triple rinsed with tap water between each sample. Clipped samples were immediately sealed with paraffin wax film in custom‐made 19‐mm‐wide by 30 cm‐long, medium‐walled borosilicate sample tubes (Corning Inc.) and placed on ice until they were moved to a freezer later in the day. Water from plant tissues was extracted by cryogenic distillation within two weeks (Kulmatiski et al., 2010). Extracted water samples were analyzed for hydrogen and oxygen isotopes on a wavelength scanned cavity ring‐down spectrometer (Picarro L‐2120*i*; Picarro Instruments). Isotope values [in delta notation (*δ*)] were converted to deuterium excess values (*δ*_e_) to control for natural isotope enrichment caused by evaporation as follows: *δ*_e_ = *δ* ^2^H – [(8 * *δ* ^18^O) + 10] (Kulmatiski et al., 2010; Mazzacavallo & Kulmatiski, 2015).

The same procedure was repeated for the 2012/2013 growing season using new plots for 10, 20, 30, 50 and 70 cm depths. Deeper (i.e., 100 cm) plots were not included because they are more difficult to perform and almost no tracer was absorbed from these depths in the 2010/2011 season.

During the mid‐season 2013 sampling, 1 g ^15^NH_4_^15^NO_3_ (0.34 mg N at 99 atom % ^15^N) was dissolved in each 1 L of 70% ^2^H_2_O tracer solution. One‐week after injections, green plant tissue samples were collected from target species in each plot. Clippers were triple rinsed with tap water between samples. Tissues from one to several individuals were placed in paper bags, air‐dried, ground, and analyzed for total N and ^15^N/^14^N ratios by continuous‐flow, direct combustion, and mass spectrometry using a Europa Scientific SL‐2020 (Sercon Limited).

### 2.3 Soil moisture

Soil matric water potential was measured at 10, 20, 30, 40, 50, 75, 100, 160, and 170 cm depths using heat dissipation matric potential sensors (Campbell Scientific 229 sensors) in one plot. Prior to installation, each sensor was calibrated using an endpoint test and by taking measurements in soils from one of three appropriate depth strata (0–30, 30–60, or 60–90 cm) that were equilibrated to each of five known water potentials for 16 hr (Flint, Campbell, Ellett, & Calissendorff, 2002). Water potentials of the equilibrated soils were determined using the chilled‐mirror technique (WP4T water potential meter; Decagon Devices). In November 2009, sensors were placed in the undisturbed wall of a soil pit. Sensor readings were recorded hourly during the growing season (CR1000 datalogger; Campbell Scientific). Soil water potentials were converted to volumetric soil moisture using published soil characteristic curves (Buitenwerf et al., 2014).

### 2.4 Data analyses

To parameterize Hydrus 1D and to allow comparisons between trees and grasses, tracer uptake values were standardized as proportional values by depth (0–125 cm; Kulmatiski et al., 2010; Mazzacavallo & Kulmatiski, 2015) as follows: $\left(S_{n}-C\right)/\left(\sum_{n=10}^{150}\left({\tilde{S}}_{n}-C\right)\right)$, where *S_n_* is the mean *δ*_e_ value of subreplicate samples for a species from injection depth *n* in a plot (e.g., grass samples at 5 cm depth in the first replicate plot), ${\tilde{S}}_{n}$ were the mean *δ*_e_ value across replicate plots for a target depth (i.e., 10, 20, 50, 75, 100 cm). *C* were *δ*_e_ values of control samples. This proportion was calculated for each plant species in a plot, producing one replicate proportional uptake value for each species in each field plot.

Differences among tracer uptake rooting profiles were tested using generalized additive mixed models (GAMMs; Wood, 2004). GAMMs were used to approximate the continuous soil profiles of tracer uptake with depth using a beta likelihood with a logit link for the linear predictor (soil depth; Kulmatiski et al., 2017; Wood, 2004). GAMMs had four (five for November 2010) “knots” to allow for a smooth interpolation between the five sample depths. Models were fit either with tree and grass distributions together or separate and the model with the lowest AIC (either “all together” or “all separate”) was the model that best balances goodness‐of‐fit against parsimony. Models were fit in R (R Core Team, 2018) using the gam function from the mgcv package (Wood, 2004).

### 2.5 Water uptake

The purpose of the water uptake modeling was to translate observed differences in tracer uptake distributions into simulated differences in the depth, time, and amount of water uptake by tree and grass roots. Following the approach of Kulmatiski et al. (2020), we used Hydrus 1D, a numerical model that simulates interception, infiltration, evaporation, water flow through the soil matrix, and water uptake from a root distribution to simulate water uptake by tree and grass rooting distributions over time (Simunek, Van Genuchten, & Sejna, 2005; Zheng et al., 2018). Model simulations were first performed using a published root biomass distribution for the site (Kulmatiski et al., 2017). Hydrus 1D was initialized with observed soil moisture data. Evapotranspiration was estimated using the Penman–Monteith equation. Microclimatic data were taken from Tobin and Kulmatiski (2018). The van Genuchten–Mualem water flow model was used. Hydraulic parameters were estimated using the neural network predictions within Hydrus with model inputs being measurements of soil texture (Buitenwerf et al., 2014), observed maximum and minimum soil water contents and associated water potentials, and bulk densities reported by Buitenwerf et al. (2014). The “Feddes” root water uptake submodel parameterized for alfalfa was used. A critical stress index of 1.0 was used since root distributions were measured directly (Kulmatiski et al., 2020). Plant height was assumed to be 60 cm and leaf area was calculated by Hydrus from plant height associated with an alfalfa crop. Model predictions of soil volumetric water content using the published root biomass distribution were reasonably well correlated with observed soil volumetric water content values (observed volumetric water content = 0.96*predicted volumetric water content + 0.02, *R* ^2^ = .52, RMSE = 0.018). All parameters used for the root biomass simulation were held constant for subsequent simulations using either the tree or grass rooting distributions defined by the tracer experiment.

Water uptake per depth strata values were converted to a per cm basis by dividing by the depth increment in the strata (i.e., 20 mm of water uptake from the 20–30 cm depth strata was reported as 2 mm per cm in this strata). This allowed estimates of the depth at which 50% of root biomass, tracer uptake, or water uptake occurred. Values are reported as a running average of 15 cm increments to smooth their distribution with depth.

### 2.6 Hydrological niches

We defined hydrological niche in two ways (Kulmatiski et al., 2020). First, the “total water uptake index” was defined as the sum of simulated water uptake by each rooting distribution, across all depths and for each growing season. Second, the unique hydrologic niche index was defined as the sum of soil water uptake that was unique to a rooting distribution:

$$\text{unique}\;\text{hydrological}\;\text{niche}\;\text{index}=\sum_{d=1}^{125}\Delta_{\max,i,d}I_{\Delta_{\max,i,d}>0}$$

where $\Delta_{\max,i,d}=U_{i,d}-\text{Max}\left(U_{j\neq i,d}\right)$, which is the water uptake of species *i* at depth *d* minus the maximum water uptake across all species (other than species *i*) for depth *d*. Thus if $\Delta_{\max,i,d}$ is positive that means species *i* had greater water uptake at that depth than any other species. $I_{\Delta_{\max,i,d}>0}$ is an indicator function, having a value of 1 if $\Delta_{\max,i,d}$ > 0 and 0 otherwise, so for a given species the index is a sum across depths of positive $\Delta_{\max,i,d}$ values. We refer to this as an index because it isolates the effects of rooting distributions and does not include the effects of potential differences between plant types such as leaf area, stomatal conductance, or aerodynamic resistance. Values are constrained by evaporative demand and soil water availability.

## 3 RESULTS

A total of 2,760 plant water isotope samples were analyzed, 1726 samples for the 2010/2011 season, and 1,034 for the 2012/2013 season. Of 143 plant samples taken from control plots, the δ^2^H values were −65 ± 30‰ (mean ± *SD*). Only one control plant sample demonstrated a δ^2^H that was 2*SD* above the mean value. This was also the only control sample with a value greater than zero. In contrast, between 16% (70 cm) and 51% (5 cm) of samples in plots that received tracer injections demonstrated δ^2^H that were 2*SD* or more above mean control values. Consequently, there was no evidence of contamination of plant samples during sample collection or processing (Kulmatiski et al., 2010).

**FIGURE 2:**
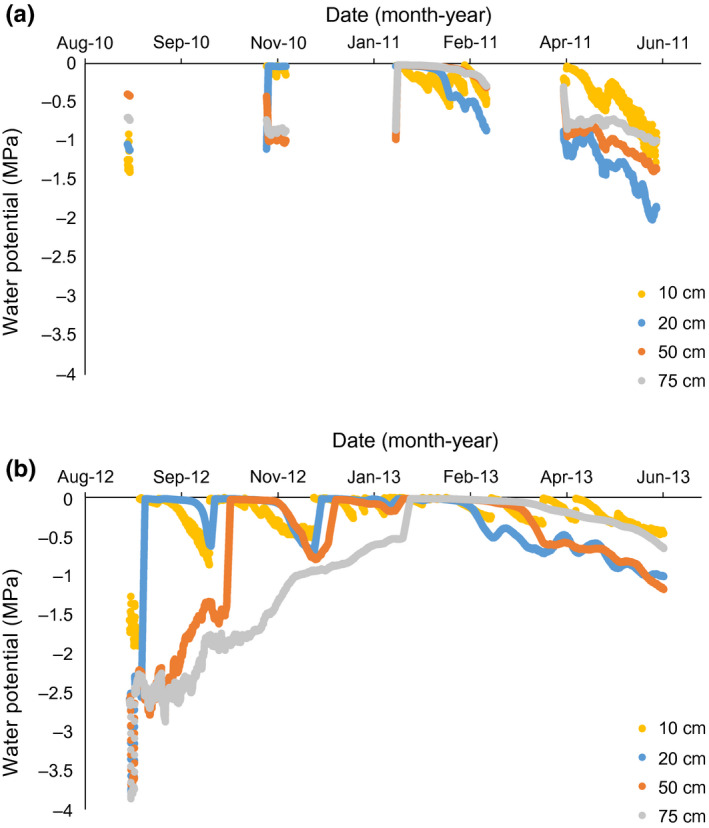
Soil water matric potentials (MPa) at selected depths during the 2010/11 (a) and 2012/13 (b) study seasons, Lower Sabie, Kruger National Park, South Africa. Measurements from one location at the study site

Soil water potentials were generally greater than −1.5 MPa (i.e., plant available) at injections depths on sampling dates (Figure 2). Soil water was generally plant available above 80 cm depths in the early‐ and late‐season samplings and plant available at all sampled depths in the middle of the growing seasons (Figure 2).

The three most frequently encountered and sampled trees were *D. cinera* (20% of all plant isotope samples), *A. nigrescens* (10%), and *Securinega virosa* (8%), with all other tree species samples representing 15% of all samples. The three most frequently encountered and sampled grasses were *P. maximum* (18%), *U. mosambicensis* (15%), *Themeda triandra* (6%), with all other grass species samples representing 9% of all samples.

### 3.1 Tracer uptake

When GAMMs were used to approximate tracer uptake profiles by depth and growth form, there was generally equal support for models that separated uptake by growth form and models that combined uptake profiles by growth form (Table 1). In other words, there was equal support for separating tree and grass rooting profiles and combining them. The exceptions were in November 2010, when there was more support for combining tracer uptake profiles and May 2013, when there was more support for separating tracer uptake profiles (Table 1).

**TABLE 1:** AIC table for models of tracer uptake by depth for six different sampling campaigns

Model	logLik	AIC	ΔlogLik	ΔAIC	*df*
Nov 2010					
All together^a^	46.2	−83.5	0	0	5
All separate	50.4	−86.3	4.2	2.9	7
Feb 2011					
All together^a^	46.6	−85.6	0	0	4
All separate^a^	46.0	−84.1	0.6	1.5	4
May 2011					
All together^a^	46.2	−83.5	4.2	0.2	5
All separate^a^	50.4	−86.3	0	0	7
Nov 2012					
All together	48.3	−90.5	2.4	5	3
All separate^a^	51.7	−95.5	0	0	4
Feb 2013					
All together^a^	18.9	−31.7	0	0	3
All separate^a^	18.1	−30.5	0.8	1.2	3
May 2013					
All together	29.8	−51.8	7.5	13.1	4
All separate^a^	37.3	−64.9	0	0	5

Note

For the “All together” model, measurements from trees and grasses were distinguished. For the “All separate” model, measurements were associated with either trees or grasses.

Abbreviations: AIC, Akaike's information criterion; *df*, degrees of freedom; logLik, log likelihood.

^a^ Indicates top model based on ΔAIC < 2 criteria.

**FIGURE 3:**
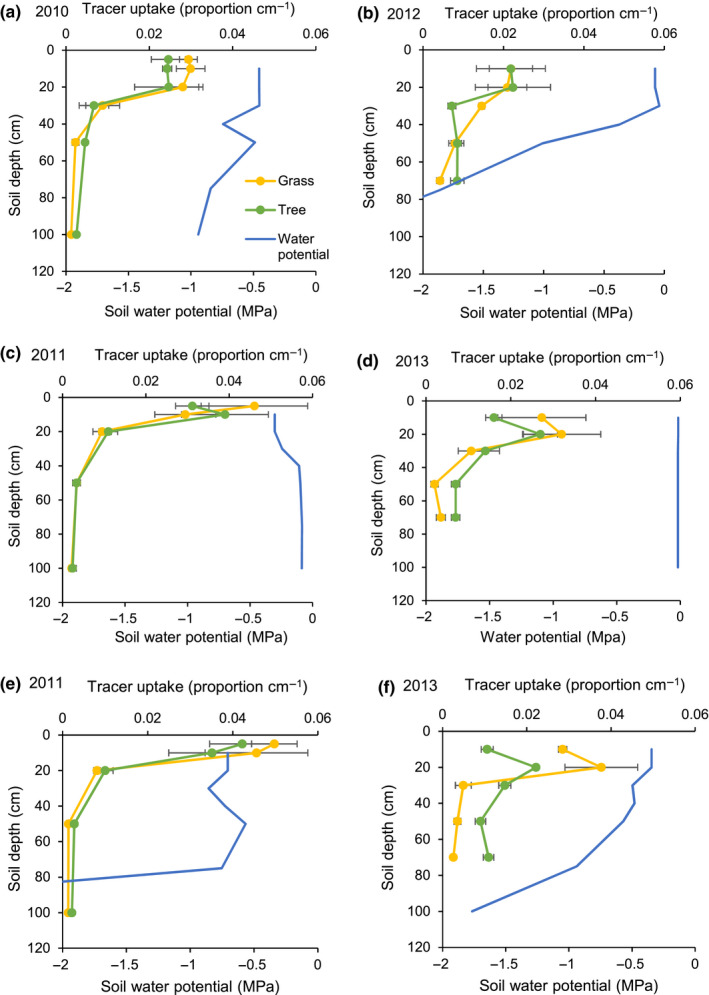
The proportion of tree and grass tracer uptake by depth for the early (a, b), mid‐ (c, d), and late‐ (e, f) 2010/2011 (a, c, e) and 2012/2013 (b, d, f) growing seasons, Lower Sabie, Kruger National Park, South Africa. A hydrologic tracer (deuterium oxide) was injected to a target depth in each of three replicate plots during each sampling date. The proportion of tracer uptake by depth was calculated for either trees or grasses separately. Error was derived from three replicate plots for each target depth. Soil water matric potential measured in one plot. Values less than ‐2.0 MPa are assumed to provide little to no plant available water

**FIGURE 4:**
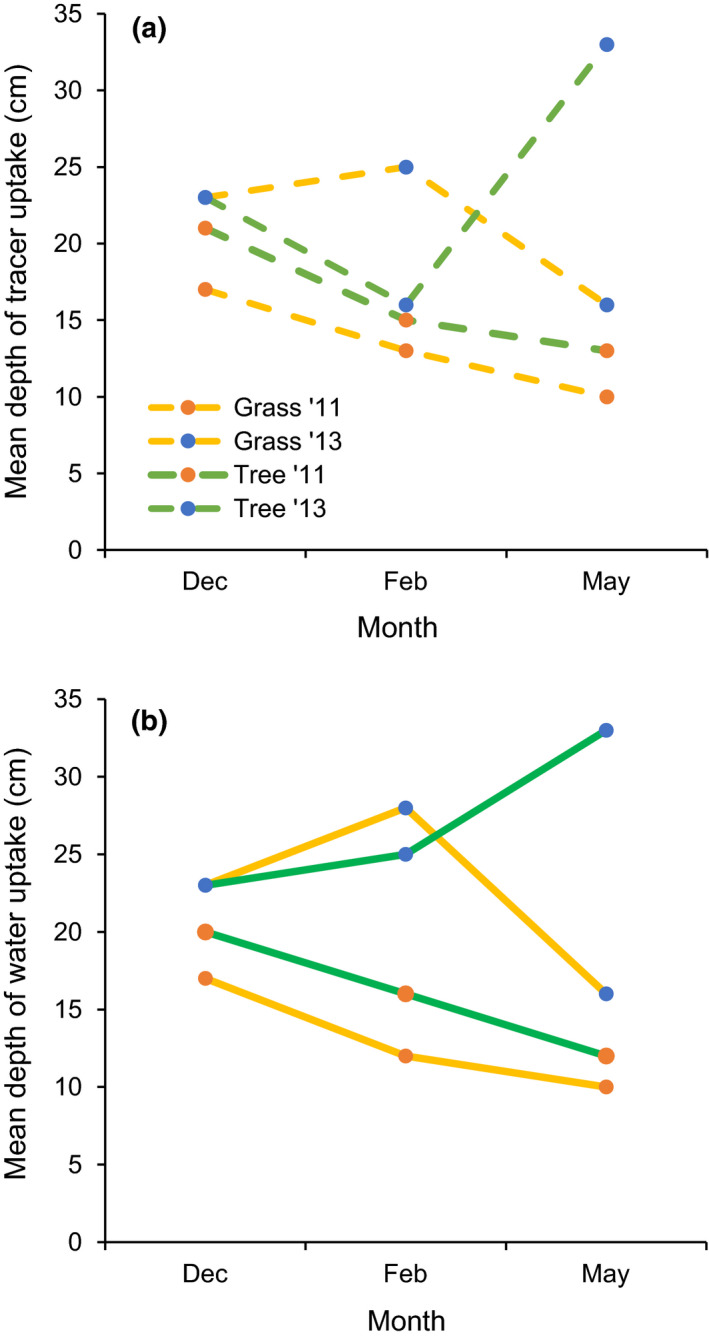
Mean depth of hydrological tracer (a) and simulated water uptake (b) for trees and grasses in the 2010/11 and 2012/13 growing seasons, Lower Sabie, Kruger National Park, South Africa. Values are cm below the soil surface and indicate the depth at which 50% of either tracer or water uptake occurred

The depth at which 50% of tracer uptake occurred (i.e., mean depth of tracer uptake) was used as an index of rooting depth. For both trees and grasses, tracer uptake was deeper in the 2012/13 season (i.e., the wetter year) than the 2010/2011 season (i.e., drier season; Table 2; Figure 2). To a lesser extent, tree tracer uptake was deeper than grass tracer uptake in both growing seasons (Figure 3). The mean depth of tracer uptake generally decreased through the growing season for both trees and grasses in the drier, 2010/2011 season (Figures 3 and 4). During the wetter 2012/2013 season, the mean depth of tree uptake increased in May and the mean depth of grass uptake decreased in May (Figures 3 and 4). These tracer uptake distributions (Figure 3) were used to parameterize the water flow model.

**TABLE 2:** The amount of water (cm) that tree and grass rooting distributions were estimated to extract (total water uptake) and the amount of water (cm) that tree and grass rooting distributions could extract in excess of the other plant growth form (unique niche)

	2010/11	2012/13
Total water uptake		
Tree	37.3	33.3
Grass	35.5	38.1
Unique niche		
Tree	0.8	0.4
Grass	0.4	1.4

Note

Water uptake was calculated by using the rooting distributions shown in Figure 3 in a water flow model. See Section 2 for further details.

### 3.2 Water uptake

**FIGURE 5:**
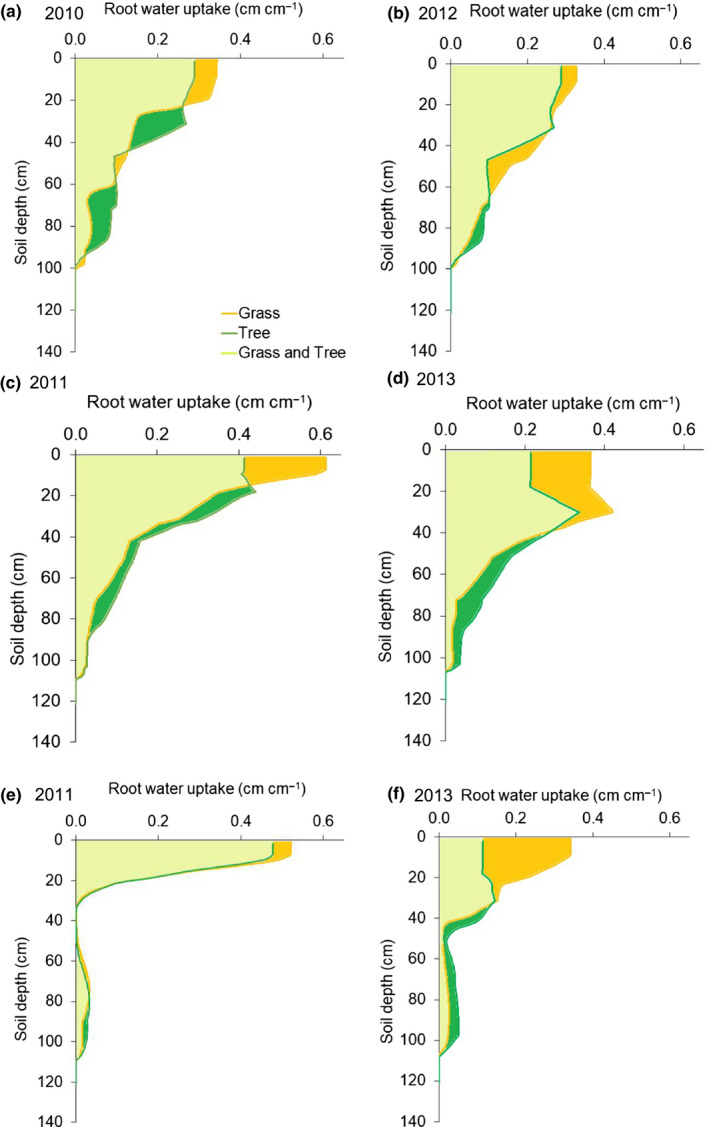
Grass and tree water uptake (cm water cm−1 soil depth) by depth for the early (a, b), mid‐ (c, d), and late‐ (e, f) 2010/2011 (a, c, e) and 2012/2013 (b, d, f) growing seasons, Lower Sabie, Kruger National Park, South Africa. Color‐filled areas indicate soil water available to both grasses and trees (light green), grasses only (yellow‐orange), and trees only (green)

Tree water uptake was slightly deeper than grass water uptake in both years (Figures 4 and 5). In the first, drier year, the mean depth of water uptake was 16 cm and 13 cm for trees and grasses, respectively (Figure 4 and 5). In the second, wetter year, the depth of 50% water uptake was 27 and 22 cm for trees and grasses, respectively (Figures 4 and 5). In the first, drier year, the tree rooting pattern was estimated to extract 5% more soil water than grasses (i.e., 37.3 vs. 35.5 cm; Table 2; Figures 4 and 5). In the second, wetter year, the tree rooting pattern was estimated to extract 13% less water than the grass rooting distribution (i.e., 33.3 cm vs. 38.1 cm; Table 2; Figures 4 and 5.

**FIGURE 6:**
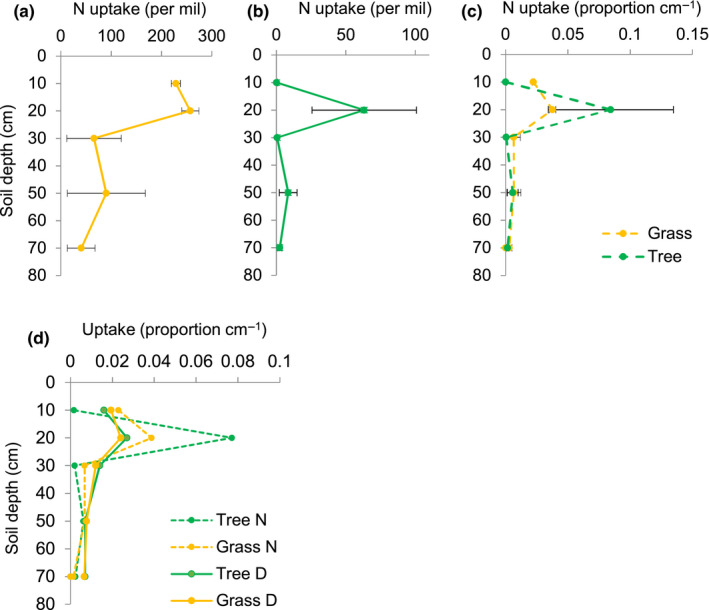
The proportion of deuterium and 15N (N) water uptake by depth for grasses and trees, Lower Sabie, South Africa, February, Higgins, Bond, and Swemmer (2013). A nitrogen tracer (15N ammonium nitrate) was injected to a target depth in each of three replicate plots during the mid‐season 2012/13 season sampling date. The proportion of tracer uptake by depth was calculated for either trees or grasses separately. Error represents the standard error in the proportion of uptake associated with the three randomly assigned replicate plots. For clarity, error bars are not shown in panel d but can be seen in Figures 3d and 6c

When GAMMs were used to describe ^15^N uptake by depth (proportion cm^−1^), there was equal support for the model that combined tree and grass uptake profiles (logLink = 87.0; AIC = −168.0, *df* = 3) and the model that separated tree and grass uptake profiles (logLink = 86.4; AIC = −168.7, *df* = 2; Figure 6). Control δ^15^N values were 3.4 ± 2.1‰ (mean ± *SD* for four samples). In plots receiving tracer, the mean ^15^N values of plant tissues were 63 ± 90‰. The mean depth of ^15^N uptake was 15 cm and 18 cm for trees and grasses, respectively. Most ^15^N tracer uptake by trees occurred from the 20 cm plots. In contrast, grasses absorbed similar amounts of ^15^N from 10 and 20 cm plots.

When GAMMs were used to compare water and ^15^N uptake by trees and grasses, there was the greatest support for a model that separated tree ^15^N uptake from all other uptake patterns (i.e., tree water uptake, grass water uptake, and grass ^15^N uptake; logLik = 159.3, AIC = −308.2, *df* = 5), though there was not more support for this model than the “All together” model (logLik = 156.4, AIC = −306.9, *df* = 3). There was more support for both of these models than the “All separate” model (logLik = 158.1, AIC = −302.8, *df* = 7).

## 4 DISCUSSION

Trees and grasses demonstrated similar shallow patterns of tracer uptake, particularly in the first study season. Such large overlap in rooting distributions is thought to preclude species coexistence. However, we found that slightly deeper root distributions provided trees more total soil water than shallower grass root distributions in the first study season, but not in the second study season. Further, trees and grasses both demonstrated depths and times at which each rooting distribution could extract more soil water than the other (Figure 5). Differences in total water uptake and unique hydrological niche sizes were not large, but they suggested that tradeoffs in rooting strategies can be expected to contribute to tree and grass coexistence because (a) competitive advantages change over time and (b) plant growth forms always have access to a soil resource pool that is not available to the other plant growth form. Determining the ecological importance of these findings will require integrating the resource‐use differences reported here with other effects, such as differences in transpiration, water use efficiency, fire, and herbivory between trees and grasses, but results demonstrate that even small differences in rooting distributions can contribute to tree and grass coexistence in savannas.

The mean depths of tree and grass water uptake were 22 and 17 cm, respectively. This difference allowed tree root distributions to absorb 5% more water than grass root distributions in the drier year (37.3 cm vs. 35.5 cm, respectively) and 13% less water in the wetter year (38.1 cm vs. 33.3 cm, respectively). This occurred because precipitation patterns in the drier year allowed deep soils (i.e., 20–100 cm) to provide a larger proportion of plant available water  . In the wetter year, more frequent rains maintained shallow soil moisture so shallow soils provided more plant available water. These results are derived from only two growing seasons, so our inference is limited, but the finding that deep roots can provide more water than shallow roots has been described in many systems (Holdo, 2013; Mazzacavallo & Kulmatiski, 2015; Ryel, Leffler, Ivans, Peek, & Caldwell, 2010; van Wijk & Bouten, 2001; Yu, Saha, & D’Odorico, 2017). Further, results provide field support for a simulation study which predicted that stochastic climate conditions will allow coexistence of species with small differences in rooting distributions (Holdo, 2013).

Trees and grasses both demonstrated depths and times at which one rooting distribution could extract more soil water than the other (Figure 5). For example, in the drier year, when tree root distributions could extract more water than grass root distributions, grasses had a unique hydrological niche of 0.4 cm (i.e., the sum of water uptake in the yellow‐shaded areas in Figure 5a,c,e). In the wetter year, when grass rooting distributions could extract more soil water than tree root distributions, trees had a unique hydrological niche of 0.4 cm. Thus, both trees and grasses had access to soil water resources that could maintain their growth even as subdominant growth forms. These niches represented less than 2% of total water uptake, but assuming 0.4 g C can be fixed per mm of water and a specific leaf area of 250 cm^2^/g, these unique hydrological niches would allow roughly 2 g C m^−2^ year^−1^, 4 g dry biomass per m^2^ year^−1^, or 10% ground cover (Huxman et al., 2004; Knapp, Ciais, & Smith, 2017). Of course, the amount of water provided by the unique hydrological niche would decrease with the relative abundance of a plant growth form. For example, in a plant community with 20% tree cover and 80% grass cover in a season with typical rainfall, grasses would have unique access to enough water to grow 8% leaf area. Alternatively, this water could be used to maintain existing biomass (Ryel et al., 2010). This water should be in addition to any other soil water for which a plant's roots could compete. For example, under the assumption of size symmetric competition, plants would have access to a proportion of the shared water resources equivalent to that plant's abundance, as well as any water in the unique hydrological niche (Cahill & Casper, 2000; Raynaud & Leadley, 2005; Rewald & Leuschner, 2009).

Water uptake was estimated for a stereotypical plant monoculture with a fixed leaf area that was assigned either the observed tree rooting distribution or the observed grass rooting distribution. This approach was used to isolate the effects of rooting distribution from other plant traits, such as leaf area, stomatal conductance, or plant aerodynamic resistance. The “unique hydrological niche” as defined here is the amount of water that one rooting distribution was estimated to extract in excess of the other rooting distribution (Kulmatiski et al., 2020). Our assumption of size symmetric root competition used with this approach appears reasonable because even though plants are likely to differ, for example, due to differences in root water potential, root asymmetric competition does not appear to have a large effect on resource uptake (Raynaud & Leadley, 2005; Rewald & Leuschner, 2009). While the approaches used here appear to provide reasonable estimates of resource uptake, future efforts aimed at integrating the effects of competition for soil water, water use efficiency, root water potentials, leaf area, and stomatal conductance can be expected to produce more refined estimates of water uptake by species, depth and time.

The mean depth of tracer uptake differed more between the dry and wet years (15 cm vs. 23 cm mean uptake depth) than between trees and grasses (22 cm vs. 17 cm mean uptake depth). Deeper tracer uptake in a wetter year is consistent with the idea that both trees and grasses foraged for soil water (Kulmatiski & Beard, 2013). In a drier year, plant available water is likely to be most consistently available at “mid‐depths” (e.g., 30–60 cm) because shallow soils will often be dry due to evaporation and transpiration and precipitation may not be sufficient to recharge very deep (e.g., 75+ cm) soils. In these conditions, deeper roots (i.e., 30–60 cm) will provide trees with more water. In contrast, in a wetter year, shallow soils will more often be wet and provide more water and at the same time more water is available deeper into the soil, encouraging deeper growth by all roots. The fact that both trees and grasses foraged in deeper soils in a wet year, but maintained niche separation, with trees maintaining deeper roots than grasses, suggests that tree and grass roots sense and avoid each other (de Kroon, Visser, Huber, Mommer, & Hutchings, 2009).

With sampling occurring during one relatively wet and one relatively dry year, inference is limited, but there appeared to be important within‐season differences in tree and grass rooting patterns. In the drier year, both trees and grasses demonstrated a very similar decrease in the mean depth of rooting across the growing season, though mean tree root depth was consistently deeper than mean grass rooting depth. In the wetter year, however, mean tree rooting depth increased through the season while mean grass rooting depth decreased at the end of the season. It is possible that a more flexible rooting strategy and greater resource storage capacity allows trees to make better use of late‐season and deeper soil resources (Ryel et al., 2010; Yu et al., 2017). It is interesting to note that deeper roots provided trees with more water in a dry year but not in a wet year, even though both trees and grasses demonstrated deeper root distributions in a wet year.

^15^N uptake demonstrated another axis of niche partitioning. Both trees and grasses absorbed the most ^15^N from 20 cm depths, though grasses absorbed more ^15^N from 10 cm depths than trees. Patterns of water and ^15^N uptake suggested that tree roots foraged independently for water and N (Bakhshandeh, Kertesz, Corneo, & Dijkstra, 2016; Kulmatiski et al., 2017; van der Heijden et al., 2015). Presumably, this reflects an ability of trees to manipulate the abundance and activity of nitrogen transporter proteins in root membrane cells (Laugier et al., 2012). Grass uptake of N, however, was similar to grass uptake of water, suggesting less regulation of N uptake by grasses than trees. This adds to a growing body of research suggesting that woody plants have more dynamic root activity that responds to resource availability (Dodd, Lauenroth, & Welker, 1998; Dubbert & Werner, 2019; Göransson, Fransson, & Jönsson‐Belyazid, 2007; Guderle et al., 2018).

The fact that the tree root distributions could extract more water than grass root distributions in the drier year (587 mm) and in a drier site (450 mm; Mazzacavallo & Kulmatiski, 2015), but not in a wetter year (866 mm) suggests that this site may be at a climatic boundary. This outcome has been predicted for sites where precipitation events are large enough for deep (>30 cm) percolation and uncommon enough that shallow soils become too dry for water uptake (Holdo, 2013; Ryel et al., 2010; van Wijk & Bouten, 2001). Interestingly, our research suggests that this climatic boundary occurs between 600 and 800 mm precipitation, which is similar to the precipitation level at which canopy closure appears to be possible across Africa (Sankaran, Ratnam, & Hanan, 2004). If tree rooting distributions can extract more water than grasses in drier conditions (<800 mm), then tree dominance in drier savannas must be controlled by other factors, such as water use efficiency, total water availability, fire, and herbivory (Case & Staver, 2017; Sankaran et al., 2004; Staver, Archibald, & Levin, 2011). Conversely, shallow roots may allow grasses to compete with trees in mesic conditions where trees would otherwise quickly overtop grasses (Ludwig, Dawson, Prins, Berendse, & Kroon, 2004; Pierce, Archer, Bestelmeyer, & James, 2019; Riginos, 2009). Experiments in Kruger National Park support these ideas. Grasses have been found to suppress trees more in wetter years (February et al., 2013). Tree/grass niche partitioning appears to decrease with precipitation suggesting that deep roots provide less advantage to trees in wetter sites (Holdo, Nippert, & Mack, 2018; Yu et al., 2017). Together, these results are consistent with the idea that deeper roots provide trees with an advantage in drier savannas and shallow roots provide grasses with an advantage in mesic savannas.

Many plant growth factors, such as fire and herbivory, affect tree and grass coexistence (Staver et al., 2011). For example, because they are more water use efficient, grass production is more responsive to available water and higher grass biomass can result in greater tree suppression by fire. These nonequilibrium processes have gained attention, but we provide an example of how even small differences in vertical rooting distributions over time may contribute to tree and grass coexistence.

## CONFLICT OF INTEREST

The authors have no conflicts of interest to declare.

## AUTHOR CONTRIBUTION

**Andrew Kulmatiski:** Conceptualization (equal); Data Curation (equal); Formal analysis (equal); Funding acquisition (equal); Investigation (equal); Methodology (equal); Project administration (equal); Resources (equal); Supervision (equal); Validation (equal); Visualization (equal); Writing‐original draft (equal); Writing‐review & editing (equal). **Karen H. Beard:** Conceptualization (equal); Funding acquisition (equal); Investigation (equal); Methodology (equal); Writing‐original draft (equal); Writing‐review & editing (equal). **Martin C. Holdrege:** Data Curation (supporting); Formal analysis (supporting); Investigation (supporting); Project administration (supporting); Writing‐review & editing (supporting). **Edmund C. February:** Conceptualization (equal); Data Curation (equal); Formal analysis (equal); Investigation (equal); Methodology (equal); Resources (equal); Writing‐review & editing (equal).

## Data Availability

Data used in this manuscript will be permanently archived and publicly accessible at USU Digital Commons (https://doi.org/10.26078/4myt‐sn42).
